# Validity and Reliability of Teleconference-Based Goniometry for Measuring Ankle and Great Toe Joint Range of Motion

**DOI:** 10.7759/cureus.81964

**Published:** 2025-04-09

**Authors:** Kathleen C Cordell, Benjamin K Wilke, Edward T Haupt, Nicolas M Dohse, Thomas G Berger, Michael G Heckman, Glenn G Shi

**Affiliations:** 1 Exercise and Sport Science, University of Georgia, Athens, USA; 2 Orthopaedic Surgery, Mayo Clinic, Jacksonville, USA; 3 Physical Medicine and Rehabilitation, Mayo Clinic, Jacksonville, USA; 4 Biostatistics, Mayo Clinic, Jacksonville, USA

**Keywords:** ankle, great toe, hallux, orthopedics, physical exam, range of motion, telemedicine

## Abstract

Introduction

Many studies focused on the positive impact of virtual visits during the COVID-19 pandemic. In the post-pandemic era, research exploring telemedicine's impact on the orthopedic physical examination, particularly for the lower extremities, continues to expand. This research evaluates the validity and reliability of telemedicine measuring ankle and great toe range of motion (ROM).

Methods

In this prospective study, 59 ankles and 59 great toes were measured by a researcher trained by a licensed occupational therapist for goniometer use both in-person and through telemedicine. Telemedicine measurements were also made by a second researcher. Mean differences and intraclass correlation coefficients (ICCs) were used to assess validity by comparing the ROM between in-person and telemedicine ROM measures. ICCs were used to evaluate reliability by assessing agreement in telemedicine ROM measures between the two different researchers.

Results

In the evaluation of validity, in-person and telemedicine measurements for both ankle dorsiflexion and plantarflexion revealed ICCs of 0.81, with mean differences equal to -0.64 and -0.93 degrees, respectively. In-person and still-shot photography for ankle dorsiflexion had an ICC of 0.83 and plantarflexion had an ICC of 0.82, with respective mean differences of -1.39 and -0.24 degrees. Hallux extension measurements between in-person and telemedical visits had an ICC of 0.87 (mean difference: 2.34 degrees), whereas still-shot showed an ICC of 0.86 (mean difference: 2.58 degrees). Hallux flexion had an ICC of 0.93 (mean difference: -0.98 degrees) for in-person and telemedical visits and an ICC of 0.95 (mean difference: -1.51 degrees) for in-person and still-shot measures.

Conclusion

Ankle and great toe joint ROM can be measured effectively during an orthopedic telemedicine visit equivalent to that of an in-person measurement.​ This supports the idea that a virtual physical ROM exam can increase efficiency for both patients and providers.

## Introduction

Telemedicine is a virtual platform that allows patients to receive remote medical care from a considerable distance. This serves as an alternative to in-person healthcare visits as it provides increased access to healthcare that is efficient and cost-effective [1]. Various countries have become acquainted with this platform, successfully diagnosing fractures and other musculoskeletal conditions [1]. Additional specialties have adopted telemedicine comprising a large proportion of visits in psychiatry, endocrinology, neurology, and internal medicine [2]. Wider acceptance and incorporation can also be attributed to improvements in technology that have increased the accessibility and quality of care that is available digitally [3]. While the landscape continues to develop, research is beginning to focus on specific advantages and disadvantages of the virtual format compared to in-person patient encounters [3]. One area under review is the physical exam.

Amid the COVID-19 pandemic, it was difficult for physicians to examine patients considering physical distancing limitations [4]. Clinicians relied on teleconferences to mitigate this obstacle. The physical examination is an important component of orthopedic care and contributes to making the initial diagnosis and tracking patient progress throughout the treatment period. Goniometry, a key measure of joint range of motion (ROM), plays a crucial role in physical examinations. A goniometer's ease of use and accessibility have made it a standard tool in orthopedic and therapeutic assessments. ROM goniometry, both in-person and virtually, has been found to have excellent test/re-test reliability suggesting telemedical goniometry is acceptable to use clinically [5]. To date, there is limited data regarding both the validity and reliability of the virtual examination compared to the traditional in-person exam.

The purpose of this study is to evaluate the validity and reliability of measuring both the ankle and first metatarsophalangeal joint (MTPJ) ROM in the virtual setting compared to an in-person visit.

## Materials and methods

After obtaining approval from the Mayo Clinic Institutional Review Board (approval number: 19-005484), we studied 59 volunteers, 32 female and 27 male. All those included were over the age of 18 and provided informed verbal consent. Inclusion criteria include the following: volunteers to be in healthy condition and comfortably perform dorsiflexion and plantarflexion at the ankle joint and flexion and extension at the first MTPJ against gravity. The researcher's visual assessment of the criteria determined the participant's eligibility for the study. The volunteer was excluded if they were unable to perform these movements independently without pain, had reported surrounding muscle weakness, or had a previous or ongoing injury or surgery to the involved joints. Exclusion extended to participants with reported neurological or pain-related impairments, including but not limited to a history of cerebrovascular diseases, brain injury, neurodegenerative conditions, cognitive impairments, or chronic or widespread pain disorders. This exclusion criteria ensured isolation of active ROM measurements.

The study took place in an at-home setting. Every volunteer was asked to perform full dorsiflexion and plantarflexion at the ankle joint and full flexion and extension at the first MTPJ. Each joint's ROM was initially recorded in person by a research personnel trained in goniometry. The research personnel then asked the volunteers to repeat these movements through a virtual platform and again recorded this data. To determine interobserver reliability, a second research personnel (a surgical resident) recorded virtual measurements. The second researcher was blinded to the results of the measurements made by the first researcher.

In-person goniometry

**Figure 1:**
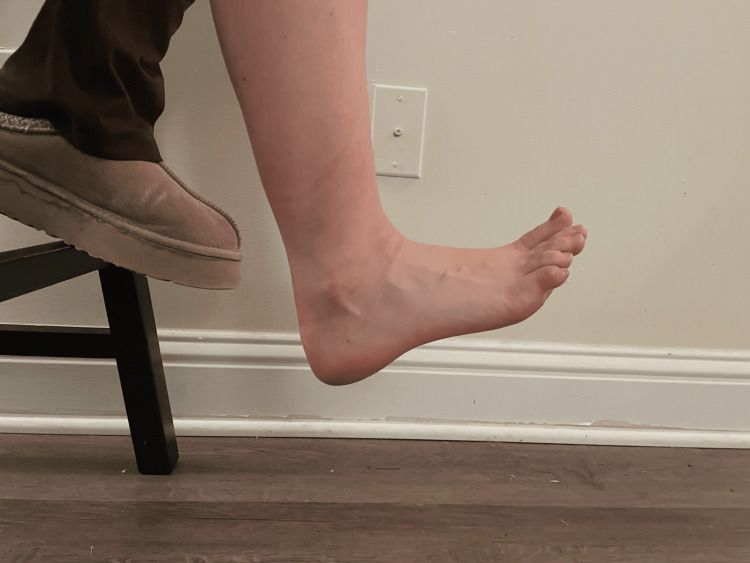
Ankle dorsiflexion A demonstration of ankle dorsiflexion was conducted by a participant in the study.

During the goniometry assessment, the volunteer was seated with their leg bent at a 90-degree angle and their foot suspended in air without touching the floor. For all ankle joint samples, the researcher measured the lateral side of the right foot. The goniometer's fulcrum was placed over the lateral malleolus, the stationary arm was aligned with the fibula, and the moving arm followed the foot's lateral border parallel to the calcaneus. The participants were instructed to perform maximum dorsiflexion upwards towards their shin (Figure 1). 

**Figure 2:**
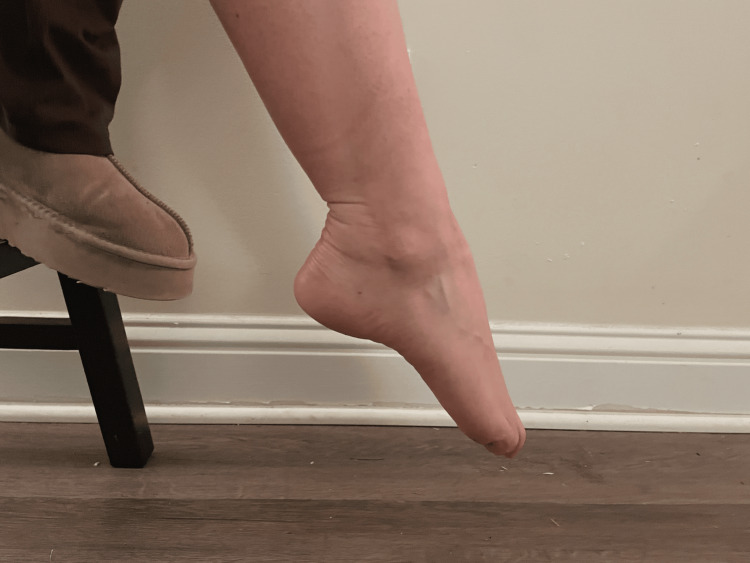
Ankle plantarflexion A demonstration of ankle plantarflexion was conducted by a participant in the study.

For max plantarflexion, the participants were instructed to point their foot downwards, away from the body (Figure 2). 

**Figure 3:**
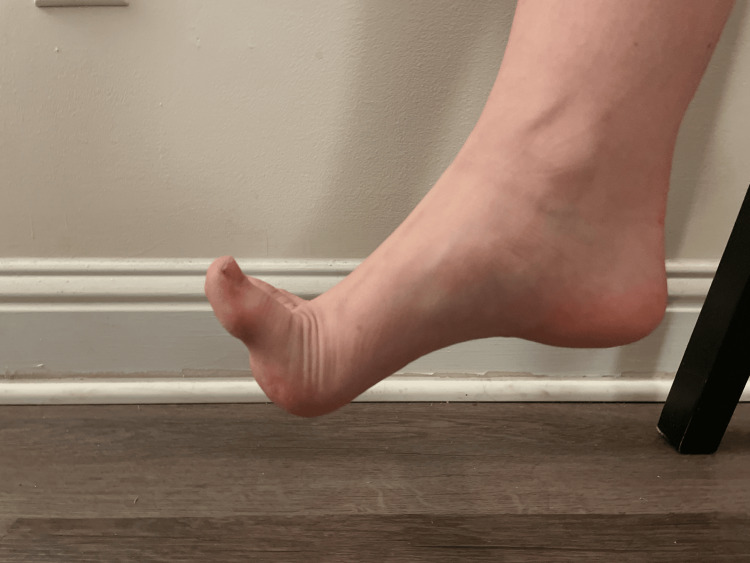
Great toe extension A demonstration of great toe extension was conducted by a participant in the study.

When measuring the MTPJ, the researcher placed the goniometer on the medial side of the foot, in line with the first metatarsal and the midline of the hallux. The ankle remained in a neutral position. The participants were instructed by the researcher to lift the toe upwards to measure the max extension (Figure 3). 

**Figure 4:**
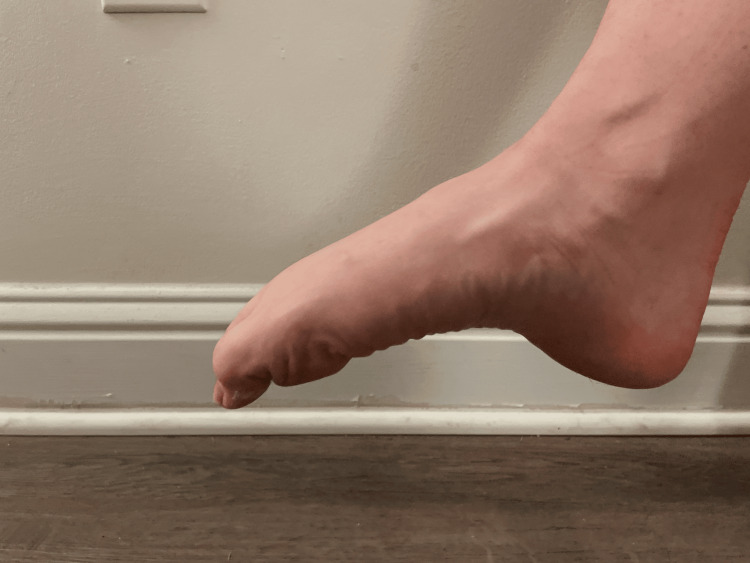
Great toe flexion A demonstration of great toe flexion was conducted by a participant in the study.

Then, the participants were instructed to curl the toe downwards to measure flexion (Figure 4). 

The instructions and foot placement were standardized for the entire study. 

Telemedical goniometry

The volunteer measurements were obtained at home to simulate a realistic virtual patient encounter with environmental and equipment variables. For ankle and great toe joint assessment, the participants wore clothing that exposed their lower leg and ankle, removed shoes and socks, and were seated in a stool or chair with feet not touching the floor, camera placed at shin level, and adequate room to perform full ROM of the joints according to standardized guidelines for telemedicine musculoskeletal examinations [6].

The telemedical simulation was conducted using the same participant placement and instructions as the in-person examination. Their right foot was suspended at a 90-degree angle, and a Logitech C920 webcam (Logitech, Newark, California, United States) was placed on the floor, 2-3 feet from the individual. The webcam was attached to a computer, and the session was recorded with attention to a 90-degree viewing angle. The volunteer was instructed to position their foot parallel to the camera where the lateral side of their foot was visible. The researcher measured the ankle joint ROM with the same goniometer from the in-person dorsiflexion and plantarflexion observations by placing the device up to the computer screen. The process was repeated for the medial side of the foot to measure great toe flexion and extension. Still-shot photographs were taken from the teleconference to allow for a second observation.

Statistical analysis

Continuous variables were summarized with the sample mean and standard deviation. In the context of this study, validity refers to the accuracy of ROM measures for telemedicine and still-shot in comparison to the gold standard of in-person, while reliability refers to the extent that telemedicine and still-shot ROM measures can be made with high agreement between different reviewers. Therefore, to assess validity, we compared ankle dorsiflexion and plantarflexion as well as hallux extension and flexion for both telemedicine and still-shot and in-person in several different ways. First, mean differences and 95% confidence intervals (CIs) were estimated. Second, intraclass correlation coefficients (ICCs) and 95% CIs were estimated. Third, Bland-Altman plots were constructed. Of note, though not an assessment of validity due to the lack of a gold-standard comparison group (i.e., in-person measures), mean differences and ICCs were also estimated when comparing telemedicine and still-shot measures.

To assess the reliability of telemedicine and still-shot ROM measures, we examined the agreement between the two observers regarding ankle dorsiflexion and plantarflexion as well as hallux extension and flexion, separately for telemedicine and still-shot, by estimating ICCs and 95% CIs. Statistical analysis was performed using R Statistical Software (Version 4.2.2; R Foundation for Statistical Computing, Vienna, Austria).

## Results

In our sample of 59 patients, an assessment of agreement in ankle and hallux ROM measures for in-person visits compared to both telemedicine and still-shot photography (i.e., validity) was conducted (Table 1).

**Table 1:** Assessment of agreement between types of measures (in-person, still-shot, or telemedicine) for ankle and toe measures SD: standard deviation; ICC: intraclass correlation coefficient; CI: confidence interval

Variable	Reviewer 1
Ankle measures (in degrees)	
Dorsiflexion	
Clinical, mean (SD)	15.75 (7.33)
Still-shot, mean (SD)	14.36 (6.81)
Telemedicine, mean (SD)	15.10 (7.40)
Still-shot minus clinical, mean difference (95% CI)	-1.39 (-2.46, -0.32)
Telemedicine minus clinical, mean difference (95% CI)	-0.64 (-1.81, 0.53)
Telemedicine minus still-shot, mean difference (95% CI)	0.75 (-0.12, 1.61)
Still-shot vs. clinical, ICC (95% CI)	0.83 (0.73, 0.90)
Telemedicine vs. clinical, ICC (95% CI)	0.81 (0.71, 0.89)
Telemedicine vs. still-shot, ICC (95% CI)	0.89 (0.82, 0.93)
Plantarflexion	
Clinical, mean (SD)	47.24 (8.08)
Still-shot, mean (SD)	47.00 (8.47)
Telemedicine, mean (SD)	46.31 (7.33)
Still-shot minus clinical, mean difference (95% CI)	-0.24 (-1.53, 1.05)
Telemedicine minus clinical, mean difference (95% CI)	-0.93 (-2.18, 0.31)
Telemedicine minus still-shot, mean difference (95% CI)	-0.70 (-1.75, 0.36)
Still-shot vs. clinical, ICC (95% CI)	0.82 (0.72, 0.89)
Telemedicine vs. clinical, ICC (95% CI)	0.81 (0.70, 0.88)
Telemedicine vs. still-shot, ICC (95% CI)	0.87 (0.79, 0.92)
Toe measures (in degrees)	
Extension	
Clinical, mean (SD)	62.73 (13.90)
Still-shot, mean (SD)	65.31 (12.53)
Telemedicine, mean (SD)	65.07 (12.54)
Still-shot minus clinical, mean difference (95% CI)	2.58 (0.77, 4.39)
Telemedicine minus clinical, mean difference (95% CI)	2.34 (0.59, 4.09)
Telemedicine minus still-shot, mean difference (95% CI)	-0.24 (-1.51, 1.03)
Still-shot vs. clinical, ICC (95% CI)	0.86 (0.78, 0.92)
Telemedicine vs. clinical, ICC (95% CI)	0.87 (0.79, 0.92)
Telemedicine vs. still-shot, ICC (95% CI)	0.93 (0.88, 0.95)
Flexion	
Clinical, mean (SD)	26.53 (17.56)
Still-shot, mean (SD)	25.02 (17.24)
Telemedicine, mean (SD)	25.54 (16.98)
Still-shot minus clinical, mean difference (95% CI)	-1.51 (-2.95, -0.06)
Telemedicine minus clinical, mean difference (95% CI)	-0.98 (-2.71, 0.74)
Telemedicine minus still-shot, mean difference (95% CI)	0.53 (-0.95, 2.00)
Still-shot vs. clinical, ICC (95% CI)	0.95 (0.92, 0.97)
Telemedicine vs. clinical, ICC (95% CI)	0.93 (0.88, 0.96)
Telemedicine vs. still-shot, ICC (95% CI)	0.95 (0.91, 0.97)

**Figure 5:**
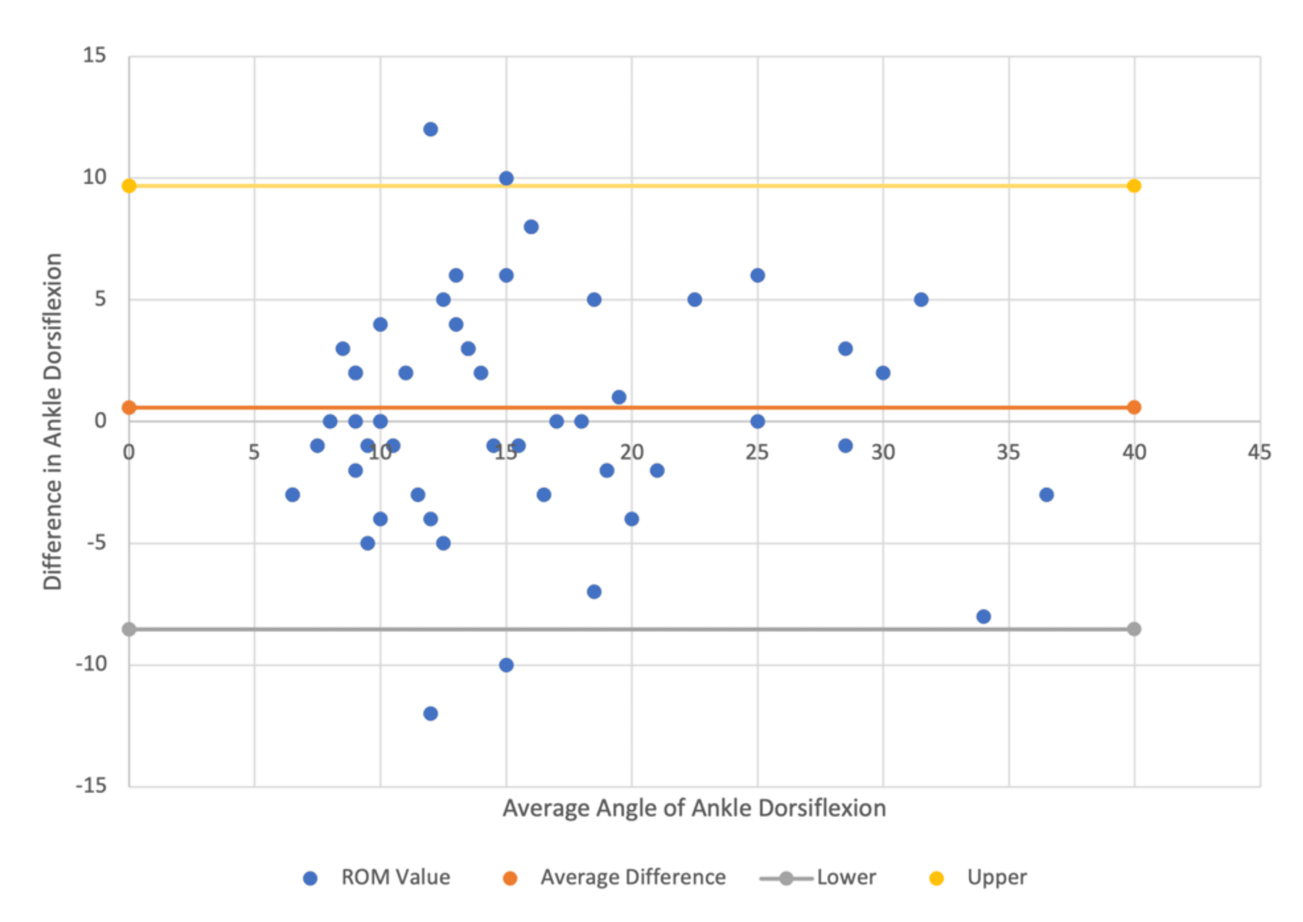
In-person vs. telemedicine Bland-Altman plot for ankle dorsiflexion data comparison within a 95% confidence interval.

The mean ankle dorsiflexion was 0.64 degrees higher for in-person compared to telemedicine measurements, with an ICC of 0.81 (Figure 5). 

**Figure 6:**
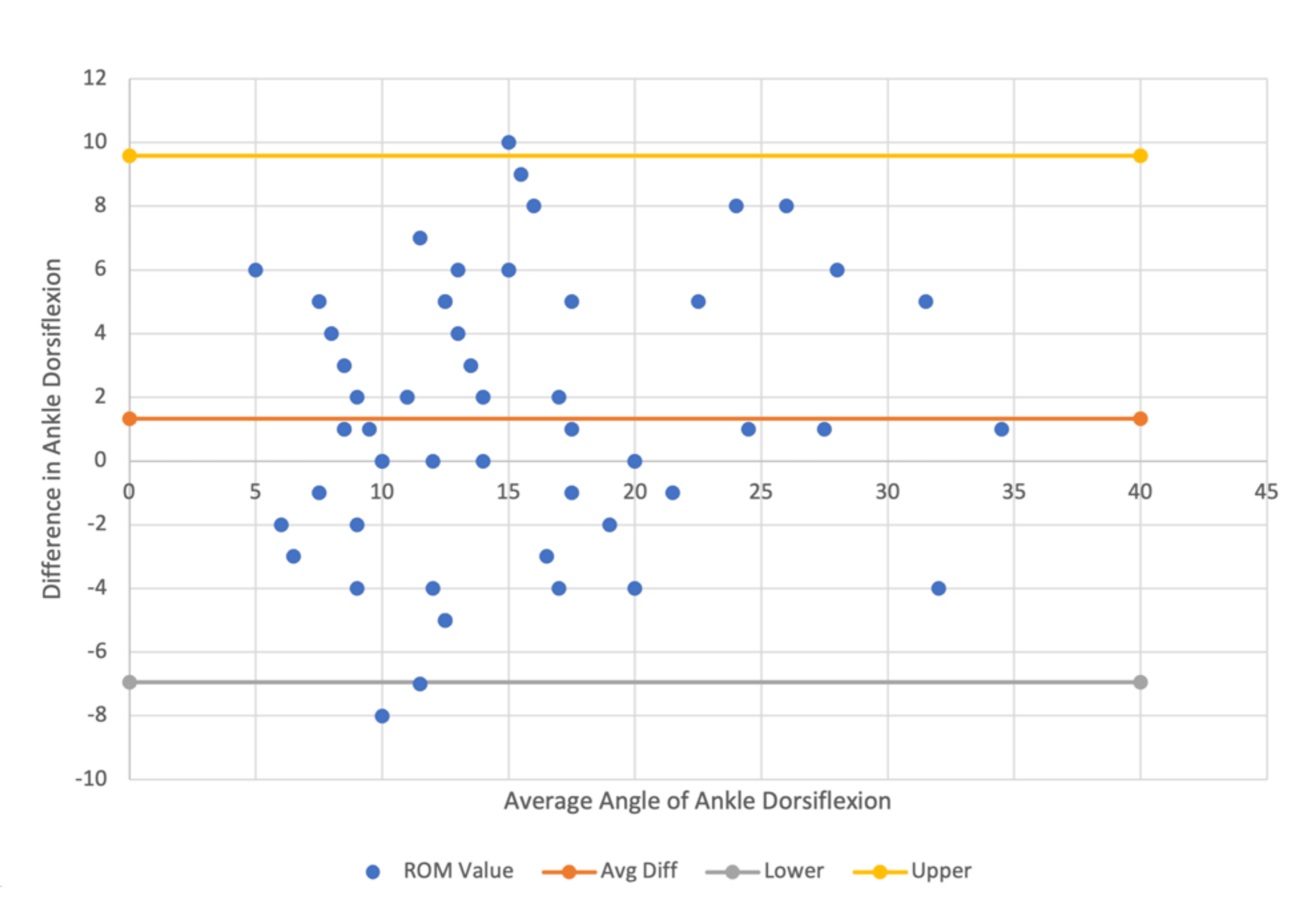
In-person vs. still-shot photography Bland-Altman plot for ankle dorsiflexion data comparison within a 95% confidence interval.

Similarly, the mean ankle dorsiflexion was 1.39 degrees higher for in-person versus still-shot photography (ICC=0.83) (Figure 6). 

Regarding ankle plantarflexion, this was 0.24 degrees higher for in-person when compared to still-shot photography (ICC=0.82) and 0.93 degrees higher when compared to telemedicine (ICC=0.81). Though not an assessment of validity, it was also of interest to directly compare telemedicine to still-shot ROM measures; agreement between telemedicine and still-shot was high for both ankle dorsiflexion (ICC=0.89) and ankle plantarflexion (ICC=0.87), with respective mean differences equal to 0.75 and -0.70 degrees.

**Figure 7:**
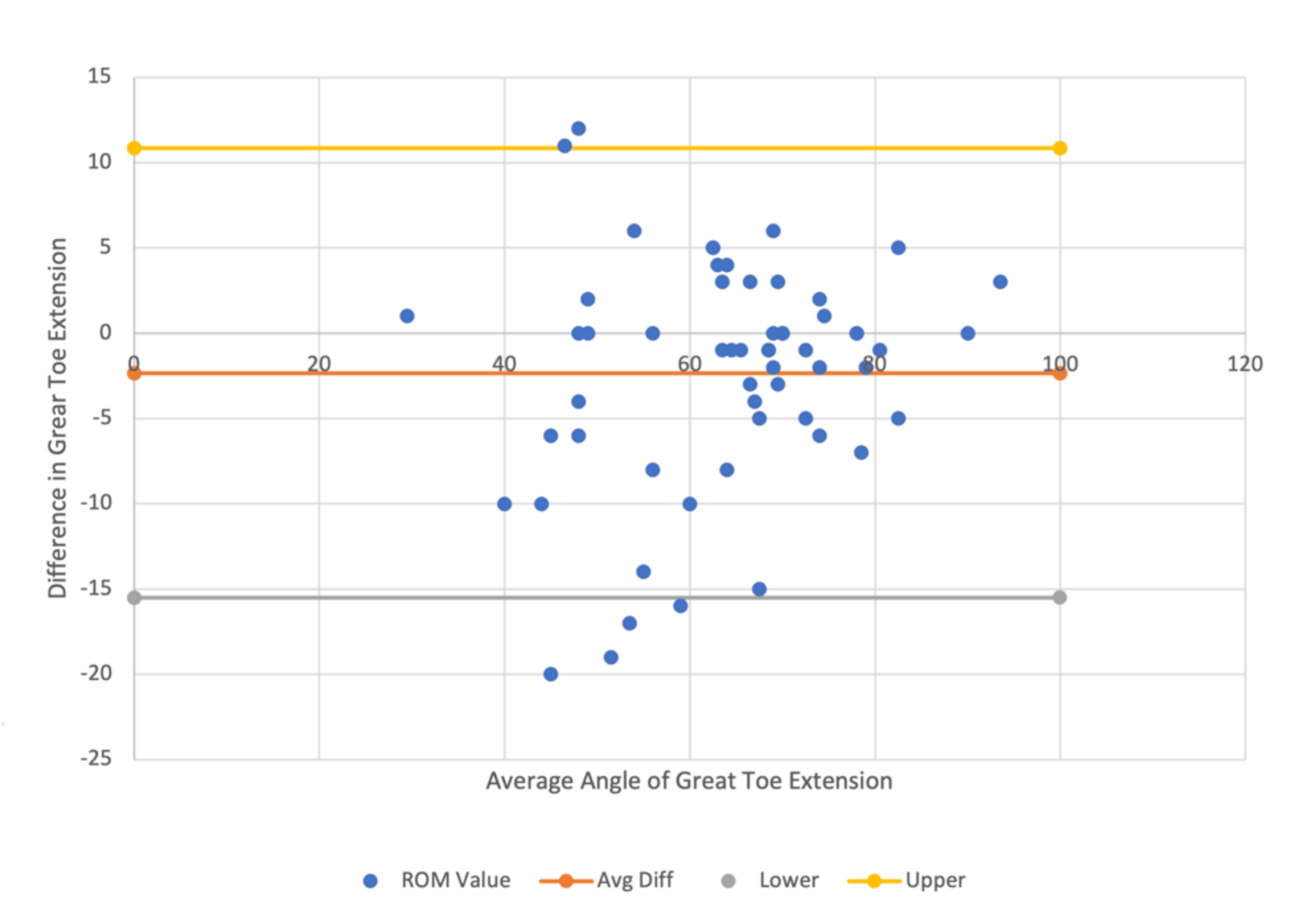
In-person vs. telemedicine Bland-Altman plot for great toe extension data comparison within a 95% confidence interval.

**Figure 8:**
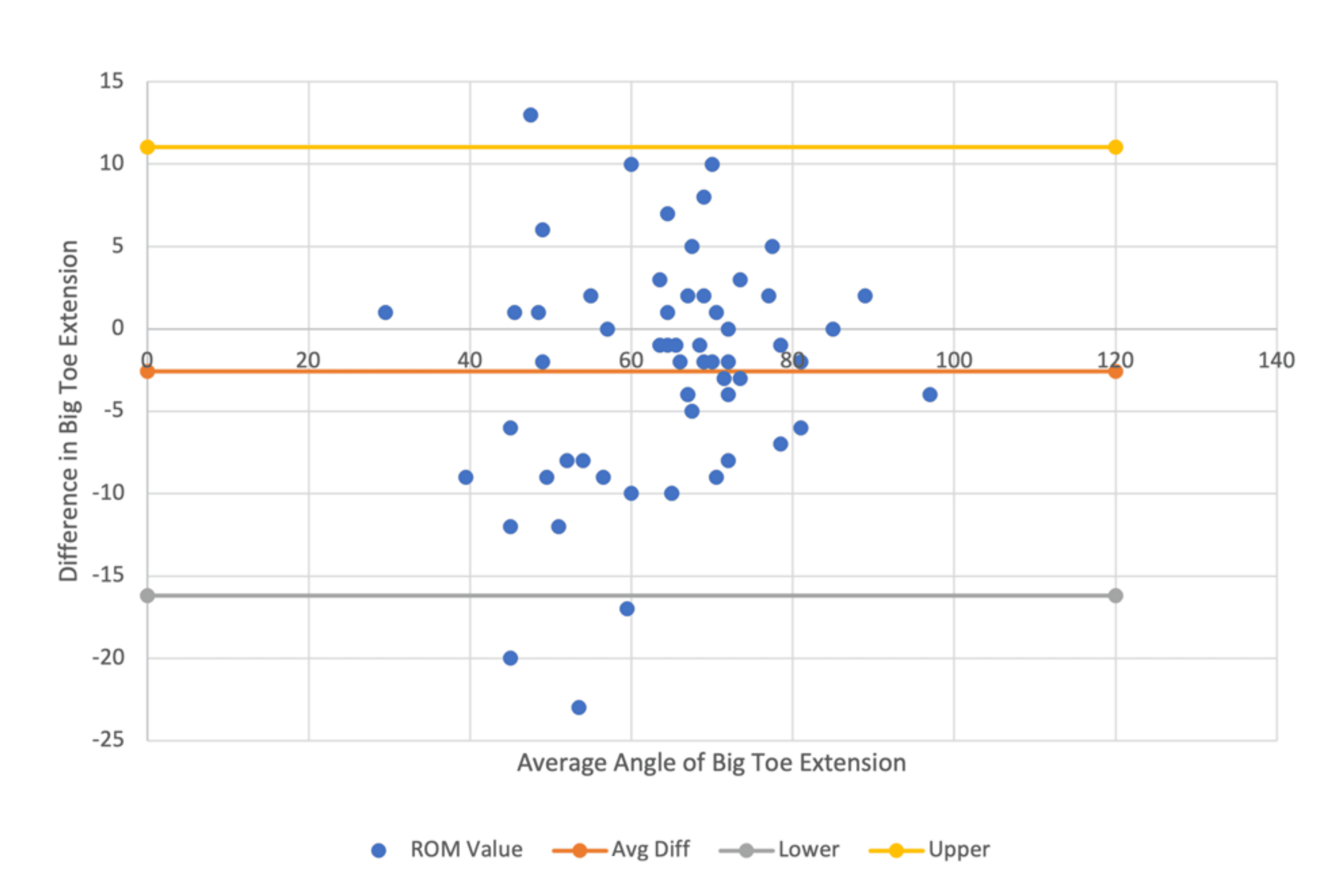
In-person vs. still-shot photography Bland-Altman plot for great toe extension data comparison within a 95% confidence interval.

In further evaluation of validity, for hallux measures, the mean extension was 2.34 degrees higher for telemedicine vs. in-person (ICC=0.87) and 2.58 degrees higher for still-shot compared to in-person measures (ICC=0.86) (Figure 7 and Figure 8). 

When assessing flexion, the mean value was 0.98 degrees lower for telemedicine compared to in-person (ICC=0.93) and 1.51 degrees lower for still-shot photography compared to in-person measures (ICC=0.95). When directly comparing the two non-gold-standard measures, agreement between measures made by telemedicine and still-shot photography was strong for both hallux extension (ICC=0.93; mean difference: -0.24 degrees) and flexion (ICC=0.95; mean difference: 0.53 degrees). 

Interobserver reliability

The interobserver agreement (i.e., reliability) in ankle dorsiflexion and plantarflexion as well as hallux extension and flexion for still-shot photography and telemedicine measures is summarized (Table 2). 

**Table 2:** Assessment of agreement between reviewers (still-shot or telemedicine) for ankle and toe measures SD: standard deviation; ICC: intraclass correlation coefficient; CI: confidence interval

Variable	Mean (SD)		ICC (95% CI)
	Reviewer 1	Reviewer 2	
Ankle measures (in degrees)			
Dorsiflexion			
Still-shot	14.36 (6.81)	13.16 (5.81)	0.86 (0.77, 0.91)
Telemedicine	15.10 (7.40)	13.75 (5.93)	0.81 (0.71, 0.89)
Plantarflexion			
Still-shot	47.00 (8.47)	45.58 (8.36)	0.71 (0.56, 0.82)
Telemedicine	46.31 (7.33)	45.73 (8.24)	0.65 (0.48, 0.78)
Toe measures (in degrees)			
Extension			
Still-shot	65.31 (12.53)	66.92 (12.06)	0.94 (0.90, 0.96)
Telemedicine	65.07 (12.54)	66.36 (12.72)	0.93 (0.89, 0.96)
Flexion			
Still-shot	25.02 (17.24)	23.51 (16.56)	0.96 (0.93, 0.98)
Telemedicine	25.54 (16.98)	24.24 (16.77)	0.95 (0.92, 0.97)

For ankle measurements, agreement in dorsiflexion between the two observers was strong for still-shot photography (ICC=0.86) and telemedicine (ICC=0.81), while agreement in plantarflexion was moderate for these two assessment methods (ICC=0.71 and ICC=0.65, respectively). When assessing inter-rater agreement in hallux ROM measures, agreement was very strong for both extension and flexion when considering both still-shot and telemedicine (ICCs from 0.93 to 0.96). 

## Discussion

In our study, compared to physically assessed measures, mean differences in ankle dorsiflexion and plantarflexion for telemedicine and still-shot measures were all less than 1.5 degrees, with high ICCs between 0.81 and 0.83. Regarding hallux measures, compared to an in-person assessment, mean differences in extension and flexion were all less than 3 degrees, with very high ICCs ranging between 0.86 and 0.95. Additionally, when considering telemedicine and still-shot measures, interobserver agreement was moderate to strong for ankle ROM measures and very strong for hallux ROM measures. This supports the ability to obtain goniometry-based ROM measurement accurately and reliably for both the ankle and the great toe via a virtual platform compared to the commonly used standard with an in-person goniometer.

One of the primary concerns regarding the implementation of telemedicine in orthopedic practices is the ability to perform an effective physical exam. Our findings, however, are consistent with the now several other studies demonstrating accuracy in the virtual examination of major joints. These studies include support for the ankle, knee, hip, shoulder, and elbow [7,8].

The findings, in combination with previously known high satisfaction rates from patients seen virtually for chronic musculoskeletal conditions, may continue to drive the field of orthopedics into the increasing implementation of telehealth [9,10], perhaps especially so in the follow-up or postoperative care setting where evaluations are more focused towards a specific diagnosis [11]. Research in this area again shows promising results with high patient satisfaction and some considering this an ideal application for safe and effective telehealth implementation [11,12].

Access to widespread telehealth services can also improve conditions for individuals with disabilities who face difficulty coordinating transportation and scheduling caregiver assistance for their routine visits [13]. Hospitals can run more efficiently and reduce direct labor costs for staff as well [14]. Following COVID-19, hospitals are finding that teleconference coverage and reimbursement are slowly adapting to the demands of service, and it is likely that payment parity will grow to encourage telemedical use [15].

Limitations of the study include a small sample size and the use of one researcher for in-person measurements, which may have introduced variability in the data and limited generalizability to broader populations. The data taken was unbiased; however, another in-person researcher could strengthen the study. The in-person evaluation was done in the same at-home setting as telemedicine, so there was no true clinical assessment. The chosen setting was most comparable to a realistic patient virtual visit, considering home set-up limitations.

In consideration of using at-home teleconference-based goniometry, patients must understand and be equipped with standardized technology protocol. Our study consisted of healthy participants who could follow instructions with ease. Future research should include diverse patient populations to assess how underlying musculoskeletal or neurological conditions may affect the functional performance of extension or flexion at the designated joints on a virtual exam. Strength and neuromuscular assessment protocols have been made available to provide ease in the virtual exam routine considering these populations [6,16]. Self-palpation by the patient can be used to assess areas of pain reproduction, and strength testing can be performed against gravity or immobilized household objects [16]. Future studies may also incorporate passive ROM using a friend or family member to facilitate joint movement.

In general, recommended devices include portable laptops or tablets with a functioning camera [6]. Additionally, smartphone digital photography provides an alternative for reliable and valid joint ROM measurements, such as for the elbow, showing promise in the accessibility of telemedicine [17]. Video and written instructions made available to a patient before a virtual visit would assist in limiting environmental variables as well.

## Conclusions

Telemedicine is an acceptable option to evaluate the ROM exam of the ankle and great toe joints, with results comparable to traditional in-person assessments. Maximum flexion and extension measures offered high ICCs and minimal mean differences between the three medians (in-person, telemedicine, and still-shot photography), indicating good validity and reliability. The findings of this study are important for the integration of telemedicine into routine orthopedic care. Virtual ROM measurements can help provide comparable care to individuals, particularly when in-person visits may prove challenging. 
